# Editorial: Advanced Microbial Biotechnologies for Sustainable Agriculture

**DOI:** 10.3389/fmicb.2021.634891

**Published:** 2021-03-25

**Authors:** Ying Ma, Miroslav Vosátka, Christopher Rensing, Helena Freitas

**Affiliations:** ^1^Centre for Functional Ecology, Department of Life Sciences, University of Coimbra, Coimbra, Portugal; ^2^Institute of Botany, Academy of Sciences of the Czech Republic, Pruhonice, Czechia; ^3^Department of Experimental Plant Biology, Charles University, Faculty of Science, Prague, Czechia; ^4^Institute of Environmental Microbiology, College of Resource and Environment, Fujian Agriculture and Forestry University, Fuzhou, China

**Keywords:** plant growth-promoting microorganisms, behavior of inoculated microorganisms, biotechnological interventions, biotic and abiotic stresses, inoculum delivery, colonization pattern, sustainable agriculture

Plant responses to various environmental or climatic stresses are immensely complex and implicate changes at the transcriptome, cellular, and physiochemical levels, consequently hindering crop growth as well as yield quantity and quality. Agriculture has been considered a complex network of plant-microbe interactions. The use of microorganisms of agricultural importance [e.g., plant growth-promoting microorganisms (PGPM)] represent a major ecological strategy for integrated agricultural practices such as nutrient addition, biological control, abiotic stress (e.g., drought, salinity, heavy metals) alleviation to minimize the use of agrichemicals (e.g., fertilizers and pesticides) in agriculture as well as to improve crop performance (Ma et al., 2011, 2016a,b). While the immense diversity of soil microorganisms represents a tremendous opportunity for selecting PGPM, the interactions with plants and the cooperative and competitive interactions among microbes themselves make it extremely challenging to determine which microbes are responsible for synergistic ecosystem functions. The essential aspects for the effectiveness of PGPM application biotechnology are the utilization of a proper inocula formulation and a suitable carrier, as well as delivery methods. Thus, considering the detrimental effects of biotic and abiotic stresses on agricultural production and food security, the development of technologies for exploring the microbial microenvironment and improving our understanding of how microbes communicate/interact with plants to enhance nutrient use-efficiency could pay substantial dividends. Therefore, this Research Topic “Advanced Microbial Biotechnologies for Sustainable Agriculture” was launched to advance our knowledge of underlying mechanisms of plant-microbe interactions and review recent progress on the relationship between the microbiome and crop productivity and health that is at the frontier of agricultural sciences, with the potential to progress and transform agricultural systems in the field.

The cogent review and synthesis embodying all the scattered information about drought and salinity stress responses and microbe-induced tolerance in crop plants were provided by Ma G. et al. They also provide insight as a means to develop an understanding of the mechanisms strongly involved in plants respond/adapt to the selected environmental stresses (e.g., drought and salinity) at the morphological, physiological, biochemical, and metabolomic levels, as well as plant-microbe interactions that confer abiotic stress tolerance in plants. So far, in most of the topics, the literature references regarding drought and salt stress are simply listed close to each other, without an integrated approach. In this review, the comparison between plant responses to drought and salinity was thoroughly highlighted and explained.

Salinity stress has been the main restraint to agriculture, limiting crop growth and productivity. Many studies have focused on utilizing PGPM to improve plant tolerance to salinity (Ma Y. et al.). It has been demonstrated that inoculation of plants with 1-aminocyclopropane-1-carboxylate (ACC) deaminase producing PGPB can enhance plant growth under salinity stress (Ma et al., 2019b). Orozco-Mosqueda et al. assessed ACC deaminase activity and trehalose accumulation of PGPB *Pseudomonas* sp. UW4, using constructed mutant strains (*acdS, treS*, or both) and a trehalose over-expressing strain (*OxtreS*). The findings indicate the synergistic action of ACC deaminase and trehalose in *Pseudomonas* sp. UW4 plays an essential role in protecting host plants against salinity stress.

To cope with drought stress, PGPM were found to secrete osmolytes to alleviate drought stress, which act synergistically with plants internal osmolytes stimulating plant growth (Paul et al., 2008). The trehalose accumulation is an adaptive mechanism in microbes in response to drought stress to protect cells and proteins from osmotic shock and desiccation (Reina-Bueno et al., 2012). Sharma et al. explore the biological importance and the role of trehalose in the tripartite symbiotic relationship between plants, rhizobia, and AMF, as well as physiological functions and molecular investigations using omics-based approaches. The review provides a critical discussion on the role of microbe-mediated trehalose accumulation in improving stress tolerance.

The use of PGPM has been considered an alternative to protect plants from diseases and improve crop productivity, reducing the amount of chemical pesticides needed (Ma Y. et al.). The ability to deconstruct fungal cell walls is a defining characteristic of fungal antagonism and anti-fungal biocontrol (Mesa-Arango et al., 2016). Schönbichler et al. explore the ability of *B. subtilis natto* to use complex fungal fruiting body and cell wall as a carbon source by secreting chitinases and proteases. The findings show that chitin does not allow bacterial growth nor induce the secretion of chitinolytic enzymes, and protease secretion might be the key mechanism for nutrient scavenging and depredating fungal cell walls by *B. subtilis natto*.

There have been thousands of scientific papers published that contribute to our knowledge on individual features of microbes, their behavior in soil, and after all their interaction with plants both in natural ecosystems and agroecosystems. Numerous papers revealed potentially huge positive effects of soil microorganisms on plant tolerance to stress and resulting ability to produce more biomass or other target yields (Reina-Bueno et al., 2012; Ma et al., 2019b; Ma Y. et al.). Nevertheless, a further step is needed to bring this knowledge closer to practice that would allow to formulate the new products and implement new biotechnologies of crop cultivation. The present Research Topic shows important advances in the understanding of the mechanisms behind plant beneficial microbial activities that help host plants cope with environmental stresses and fills the gap to translate scientific knowledge into sustainable applications.

As examples, Rocha et al. and Ferreira et al. show that the knowledge transfer to real agriculture can be feasible since there are numerous beneficial microbes that we know and have isolated and even established effective procedures for their mass production. However, the delivery systems for their large-scale applications in the field represent the most common bottleneck.

Seed coating has been considered a precise and cost-effective method to deliver microbial inoculants. A delivery system based on seed coating of various microbes seems to be economically feasible and applicable even in broad-acre agriculture (Ma et al., 2019a). As discussed in Rocha et al., there are still numerous considerable factors that hamper the wider use of microbial seed coating and in general the use of microbes as bioagents in general agriculture practice. The most important ones are the self-life of microbes after coating, their compatibility among themselves, and their ultimate efficacy when they are used in mixtures. There is also a crucial factor in production and application costs. The seed companies are not always keen to change their long-term practices and use biologicals instead of chemical treatment of their seeds (also taking into account that those two treatments are unlikely to be compatible with each other). Moreover, farmers are usually not equipped to do the seed treatment themselves and they have little incentives to ask seed companies for microbially coated seeds (charged premium price) until they see significant evidence of better-coated seed performance. Last but not least there is a general issue of final cost per hectare and especially for low-value crops like cereals where there are generally low-profit margins, it is difficult to accommodate any extra costs. Moreover, the special issue for biocontrol microbes mandates a very strict and costly registration (in particular within the EU).

Nevertheless, with proceeding soil degradation, increasing effects of global climatic change and after all growing awareness and demands for agrochemical reduction, healthy and secure food crops, the microbial seed coating technology will certainly be growing in its implementation and wider use. The transition of scientific knowledge to a real commercial application has been happening at a large scale already for some important crops and it holds a strong potential for the near future for other microbes and crops worldwide. A very good example of a fundamental science study conducted by Ferreira et al. that can be transformed into real agriculture is the testing of bacteria-based fertilizer that can alleviate iron-deficiency-induced chlorosis (IDIC). In this work, the ability of two new Fe freeze-dried fertilizer products, prepared from the filtrate cultures of *A. vinelandii* and *B. subtilis* was tested using an important soybean crop. Plants treated with *A. vinelandii* Fe fertilizer developed a dry mass comparable to that of o,o-EDDHA and the *A. vinelandii*-treated plants had higher Fe content. The results indicated that the freeze-dried product, prepared from *A. vinelandii*, represents a very promising, sustainable, and environment-friendly Fe-fertilizer alternative for application in the IDIC amendment in calcareous soils. Similar calcareous soils that are naturally alkaline or are being threatened by increasing salinity are becoming more abundant globally (Yadav et al., 2011). Also, the Fe deficiency is generally increasing in arable soils of numerous regions of the world and therefore similar fertilizers can be a biologically-based solution for that.

Interactions between plants and microbes including fungi are mediated by a chemical language containing multiple compounds, infochemicals, such as terpenes (Schmidt et al., 2017). We are slowly deciphering this communication that could be called signalomics (Mhlongo et al., 2018) to understand the interplay between environment, plants, and not only microbes but also other organisms. Phytohormones produced not only by plants but also by microbes play a crucial role in these interactions as described in a review by Kudoyarova et al.. Auxin-producing bacteria were shown to influence processes such as root elongation but both root elongation and inhibition of root elongation could be observed depending on the plant, the environment, and the dosage and the auxins. Other phytohormones include cytokinins such as zeatin riboside produced by certain bacteria also influence plant physiology but is so far even less elucidated. Other important phytohormones generated by microbes include ACC deaminase and abscisic acid (ABA). ACC deaminase lowers ethylene production but the effect of ABA accumulation is not often defined by a clear-cut physiological effect.

In a paper dealing with a related topic, Luziatelli et al. examine the effect of plant growth-promoting *Pantoea agglomerans* on the rooting of *Pyrus communis*. It could be shown that exometabolites such as indole-3-acetic acid (IAA) of *P. agglomerans* promoted adventitious rooting. Of interest is that the synergy between auxin-related compounds such as IAA and other metabolites produced by *P. agglomerans* such as cinnamic-related compounds was shown to be very delicate and concentration-dependent. As previously shown for IAA, there is an optimal concentration and more is not necessarily better. Here it was demonstrated that the optimal concentration of auxin-like products is also dependent on the simultaneous production of other yet to be defined products.

In another article, Xu et al. examine the relationship between soybean genotype, arbuscular mycorrhiza fungi, and rhizobium inoculation. The soybean genotype directly influenced the establishment of the rhizosphere fungal community and additionally, rhizobium inoculation also determined the composition of the rhizosphere fungal community. We are only at the beginning of understanding these complicated interkingdom dynamics.

In conclusion, there is great potential for near future enhancements in the use of PGPM in world agriculture. A paramount need is to bridge the gap between fundamental, applied science, and agricultural practice. As early as a possible transition of knowledge to the farmers as end-users of innovative products and biotechnologies can ensure efficient commercialization of scientific results and can also fuel new research on the way to achieve more sustainable use of natural resources and more efficient biologically/ecologically based agriculture.

## Author Contributions

YM, MV, and CR drafted the editorial text. YM and HF revised and approved the final version of the editorial text. All authors contributed to the article and approved the submitted version.

## Conflict of Interest

The authors declare that the research was conducted in the absence of any commercial or financial relationships that could be construed as a potential conflict of interest.

## References

[B1] Ma Y. Látr A. Rocha I. Freitas H. Vosátka M. Oliveira R. S. (2019a). Delivery of inoculum of *Rhizophagus irregularis* via seed coating in combination with *Pseudomonas libanensis* for cowpea production. Agronomy 9:33. 10.3390/agronomy9010033

[B2] Ma Y. Oliveira R. S. Freitas H. Zhang C. (2016a). Biochemical and molecular mechanisms of plant-microbe-metal interactions: relevance for phytoremediation. Front. Plant Sci. 7:918. 10.3389/fpls.2016.00918 27446148 PMC4917562

[B3] Ma Y. Prasad M. N. V. Rajkumar M. Freitas H. (2011). Plant growth promoting rhizobacteria and endophytes accelerate phytoremediation of metalliferous soils. Biotechnol. Adv. 29, 248–258. 10.1016/j.biotechadv.2010.12.001 21147211

[B4] Ma Y. Rajkumar M. Oliveira R. S. Zhang C. Freitas H. (2019b). Potential of plant beneficial bacteria and arbuscular mycorrhizal fungi in phytoremediation of metal-contaminated saline soils. J. Hazard. Mater. 379:120813. 10.1016/j.jhazmat.2019.120813 31254792

[B5] Ma Y. Rajkumar M. Zhang C. Freitas H. (2016b). Beneficial role of bacterial endophytes in heavy metal phytoremediation. J. Environ. Manag. 174, 14–25. 10.1016/j.jenvman.2016.02.047 26989941

[B6] Mesa-Arango A. C. Rueda C. Román E. Quintin J. Terrón M. C. Luque D.. (2016). Cell Wall changes in amphotericin B-resistant strains from *Candida tropicalis* and relationship with the immune responses elicited by the host. Antimicrob. Agents Chemother. 60, 2326–2335. 10.1128/AAC.02681-15 26833156 PMC4808153

[B7] Mhlongo M. I. Piater L. A. Madala N. E. Labuschagne N. Dubery I. A. (2018). The chemistry of plant-microbe interactions in the rhizosphere and the potential for metabolomics to reveal signaling related to defense priming and induced systemic resistance. Front. Plant Sci. 9:112. 10.3389/fpls.2018.00112 29479360 PMC5811519

[B8] Paul M. J. Primavesi L. F. Jhurreea D. Zhang Y. (2008). Trehalose metabolism and signaling. Annu. Rev. Plant Biol. 59, 417–441. 10.1146/annurev.arplant.59.032607.092945 18257709

[B9] Reina-Bueno M. Argandoña M. Nieto J. J. Hidalgo-García A. Iglesias-Guerra F. Delgado M. J.. (2012). Role of trehalose in heat and desiccation tolerance in the soil bacterium *Rhizobium etli*. BMC Microbiol. 12:207. 10.1186/1471-2180-12-207 22985230 PMC3518184

[B10] Schmidt R. Jager V. Zühlke D. Wolff C. Bernhardt J. Cankar K.. (2017). Fungal volatile compounds induce production of the secondary metabolite Sodorifen in *Serratia plymuthica* PRI-2C. Sci. Rep. 7:862. 10.1038/s41598-017-00893-3 28408760 PMC5429845

[B11] Yadav S. Irfan M. Ahmad A. Hayat S. (2011). Causes of salinity and plant manifestations to salt stress: a review. J. Environ. Biol. 32, 667–685. 22319886

